# Reversing chemoresistance in ovarian cancer: network pharmacology reveals how hydroxychloroquine/sulfasalazine duotherapy remodels tumor inflammatory–immune microenvironment

**DOI:** 10.3389/fimmu.2026.1790210

**Published:** 2026-03-26

**Authors:** Xin Hong, Xin Wen, Ting Zhang, Yihao Liu, Yu Ji, Xiaoyan Shen

**Affiliations:** 1 Department of Obstetrics and Gynecology, Peking University People’s Hospital, Beijing, China; 2 Department of Clinical Medicine, Peking University Health Science Center, Beijing, China; 3 Department of Orthodontics, Peking University School and Hospital of Stomatology and National Center for Stomatology and National Clinical Research Center for Oral Disease and National Engineering Research Center of Oral Biomaterials and Digital Medical Devices, Beijing, China

**Keywords:** chemosensitization mechanisms, chemotherapy resistance, hydroxychloroquine, maintenance treatment, ovarian cancer, sulfasalazine, tumor microenvironment

## Abstract

**Introduction:**

Chemoresistance is a key contributor of ovarian cancer (OC) mortality. Clinical observations of extended survival in OC patients with rheumatic comorbidities following anti-rheumatic treatment suggest hydroxychloroquine (HCQ) and sulfasalazine (SSZ) could act as chemosensitizers. However, how the HCQ/SSZ combination counteracts platinum–taxane resistance remains unclear.

**Methods:**

A multiomics strategy was applied, integrating transcriptomics from resistant and sensitive OC models with network pharmacology, consensus clustering, machine learning, and molecular docking, which indicated potential binding to predicted targets (e.g., SSZ–tumor necrosis factor (TNF): −6.99 kcal/mol). Validation included *in vitro* drug sensitivity assays and clinical cytokine profiling.

**Results:**

Analysis identified 26 overlapping genes as shared targets and classified patients into chemoresistant (Subtype 1) and chemosensitive (Subtype 2) subgroups, with Subtype 1 associated with protumorigenic pathway enrichment, immunosuppressive features, and poorer prognosis. A seven-hub-gene predictive signature was established. HCQ/SSZ appeared to remodel the inflammatory–immune tumor microenvironment, primarily through cytokine and nuclear factor kappa B (NF-κB) signaling. Clinical cytokine data supported a localized immunosuppressive niche, and *in vitro* evidence confirmed cytotoxic effects. Patients receiving long-term HCQ/SSZ therapy generally showed improved clinical outcomes.

**Conclusion:**

This study suggests that HCQ/SSZ may reverse chemoresistance by reprogramming the inflammatory–immune microenvironment, offering a molecular rationale for further investigation into their repurposing as chemosensitizers and maintenance therapies in OC.

## 1 Introduction

Ovarian cancer (OC) remains the deadliest gynecologic malignancy, with a 5-year survival rate of <40%, primarily due to late diagnosis and the emergence of resistance to chemotherapy (1). Platinum–taxane combinations are the cornerstone of first-line therapy; however, nearly 80% of patients relapse with drug-resistant disease, highlighting an urgent need for strategies to overcome resistance and improve survival (1, 2).

Emerging evidence reports that patients with OC and comorbid rheumatic disorders, such as sicca syndrome or dermatomyositis, may have better outcomes than those without autoimmune conditions (3). In these patients, long-term survival is associated with continuous antirheumatic therapy throughout the treatment continuum, including pretreatment, active therapy, and posttreatment maintenance. Common agents include hydroxychloroquine (HCQ) (3), sulfasalazine (SSZ), and iguratimod, which modulate inflammatory pathways that may influence oncologic outcomes. HCQ, an antimalarial and immunomodulatory drug, exhibits multifaceted antitumor effects, inducing apoptosis, suppressing autophagy, and remodeling the tumor microenvironment via toll-like receptor 9 (TLR9)/NF-κB modulation, p53 activation, and CXCR4–CXCL12 signaling alteration. Moreover, HCQ promotes vascular normalization, reprograms tumor-associated macrophages from the M2 to M1 phenotype, and activates cancer-associated fibroblasts, thereby enhancing anticancer efficacy (4). Additionally, HCQ sensitizes cancer cells to cytotoxic agents, facilitating chemotherapy success (5).

SSZ, a conventional anti-inflammatory drug, also exhibits anticancer activity. Its effects likely involve ferroptosis induction, suggesting that SSZ can chemosensitize cancer cells and reverse drug resistance (6–11). An injectable chitosan–dextran hydrogel loaded with SSZ has been shown to induce ferroptosis and macrophage reprogramming, suppressing ascites and enhancing anti-programmed cell death protein 1 (PD-1) efficacy in hepatocellular carcinoma (12).

These findings support the following hypothesis: immunomodulatory and anti-inflammatory drugs used to treat rheumatic diseases exert dual antitumor effects by chemosensitizing tumor cells and reversing drug resistance. To test this hypothesis, we applied integrative network pharmacology and molecular docking to investigate the multimodal mechanisms of HCQ and SSZ in overcoming chemotherapy resistance (13). This objective required integrating multisource and heterogeneous omics data, resolving pathway crosstalk within complex chemoresistance networks, and confirming drug-target specificity.

Here, we employ integrative network pharmacology and molecular docking to dissect the multimodal mechanisms by which HCQ and SSZ overcome platinum–taxane resistance in OC, aiming to establish a molecular foundation for repurposing these antirheumatic drugs as chemosensitizers and maintenance therapeutics.

## 2 Materials and methods

### 2.1 Study flowchart

**Figure 1:**
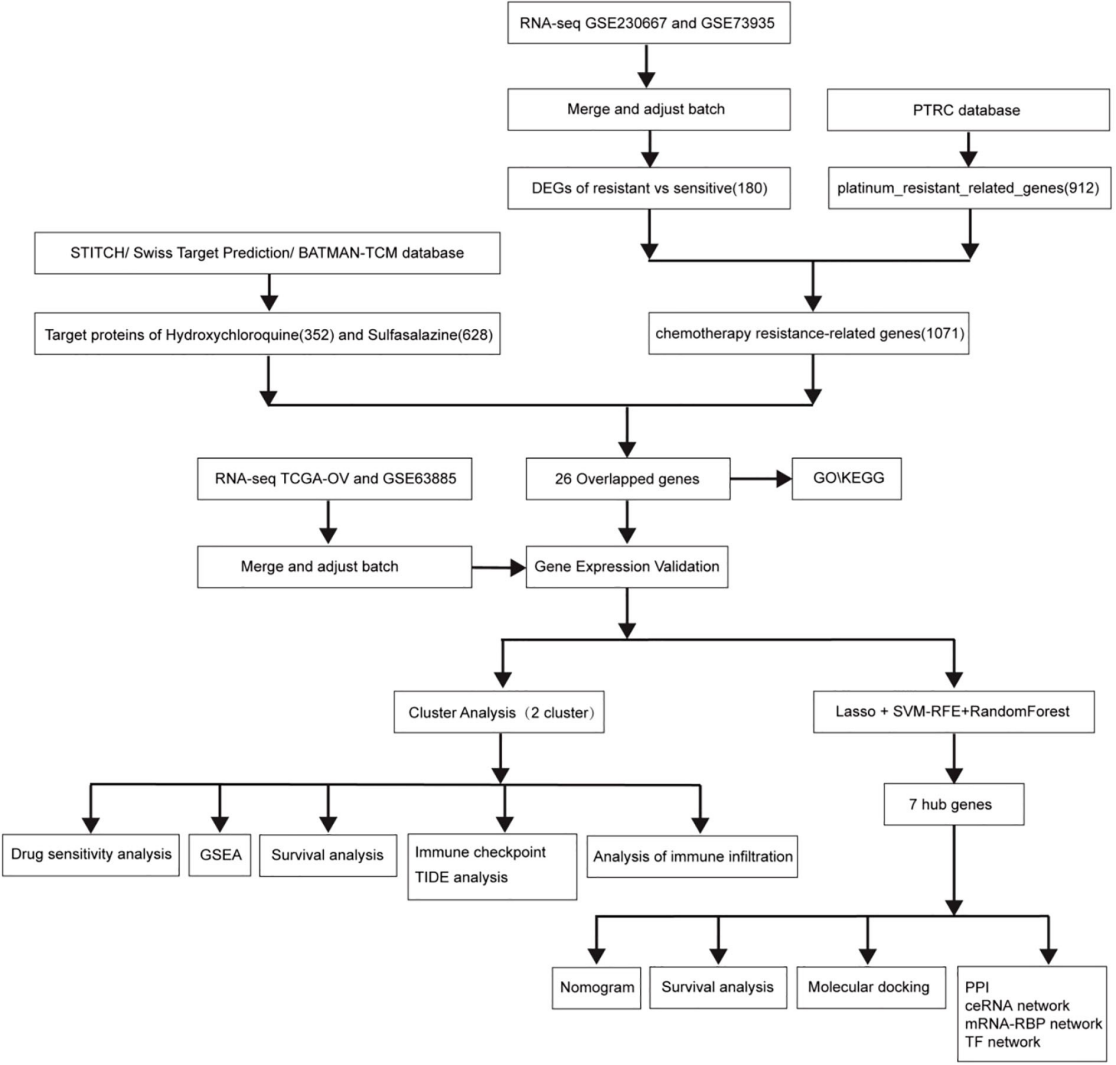
Study flowchart.

**Figure 1** illustrates the study flowchart.

### 2.2 Data sources and preprocessing

All data were obtained from public databases: Gene Expression Omnibus (GEO) and The Cancer Genome Atlas (TCGA). Three GEO transcriptomic datasets (GSE230667, GSE73935, and GSE63885) were analyzed. The dataset GSE230667 was sequenced on the GPL28038 DNBSEQ-G400 (Homo sapiens) platform and contains a total of 2 samples, including 1 paclitaxel-resistant and 1 paclitaxel-sensitive sample. The dataset GSE73935 is based on GPL13667 [HG-U219] Affymetrix Human Genome U219 Array, containing 113 samples, from which the paclitaxel-resistant samples of the A2780 cell line (GSM1906472-GSM1906477) and the control samples (GSM1906484-GSM1906486) (6:3) were selected for analysis in this study. The dataset GSE63885 is based on GPL570 [HG-U133_Plus_2] Affymetrix Human Genome U133 Plus 2.0 Array, from which 101 ovarian cancer samples were included in this study. Additionally, data from the TCGA ovarian serous cystadenocarcinoma (TCGA-OV) data, including 429 OC tissues with whole-genome expression profiles (transcripts per million) and clinical metadata, provided comprehensive molecular and phenotypic characterization. GSE230667 (1 paclitaxel-resistant and 1 paclitaxel-sensitive) was merged with GSE73935 (6 paclitaxel-resistant samples and 3 control samples) to obtain a combined paclitaxel resistance-related dataset containing 7 resistant samples and 4 sensitive samples, and TCGA-OV and GSE63885 were integrated as a training set (530 OC samples). Batch effects were corrected using ComBat (sva R package, v3.21.0) and validated using principal component analysis(**Supplementary Figure 1**). As only publicly available datasets were used, the study was considered exempt from ethical review. Chemoresistance-related genes were defined as the union of platinum-resistant genes (14) and paclitaxel resistance–associated genes.

### 2.3 Drug molecular target screening

Candidate molecular targets for HCQ and SSZ were identified from three pharmacological databases: STITCH (interaction score ≥ 0.7) (15), SwissTargetPrediction (16), and BATMAN-TCM (adjusted P < 0.05 with a score ≥ 5) (17). Targets for HCQ were consolidated using a union operation to generate a comprehensive target set; the same approach was applied for SSZ. This ensured maximal coverage of drug–protein interactions while considering database-specific prediction algorithms and reducing selection bias.

### 2.4 Analysis of differentially expressed genes

To identify the core targets associated with paclitaxel resistance, the R package limma (version 3.50.3) was used to detect differentially expressed genes (DEGs) between paclitaxel-resistant and -sensitive groups. DEGs were screened using thresholds of absolute log2 fold change (|log2FC|) > 1 and adjusted P < 0.05 and were defined as paclitaxel resistance–associated genes for subsequent mechanistic investigations.

### 2.5 Gene Ontology and Kyoto Encyclopedia of Genes and Genomes pathway enrichment analyses

We conducted functional enrichment analysis of Gene Ontology (GO) terms and Kyoto Encyclopedia of Genes and Genomes (KEGG) pathways to determine the biological significance of identified DEGs. While the GO analysis covered biological process (BP), molecular function (MF), and cellular component (CC) terms, the KEGG pathway analysis identified enriched signaling pathways and metabolic networks. Both analyses were performed using the R package clusterProfiler (version 4.2.2), with Benjamini–Hochberg-adjusted P values <0.05 defining significance.

### 2.6 Consensus cluster analysis

Consensus clustering was applied iteratively on subsampled datasets to assess cluster stability and guide parameter selection by leveraging subsampling-induced variability. The integrated dataset of 530 OC samples from TCGA-OV and GSE63885 was clustered using the R package ConsensusClusterPlus (v1.58.0) with target genes as features. The algorithm ran 1,000 iterations with k (cluster number) = 6 to ensure robust clustering through repeated subsampling and cluster reassignment.

The Kaplan–Meier (KM) curve was plotted using the ggsurvplot function from the R package survminer (0.4.9). The log-rank test was calculated using the pchisq function from the R package stats (4.1.2), with all statistical parameters set to the R package defaults.

### 2.7 Drug sensitivity analysis

Drug sensitivity across OC subtypes was analyzed using half-maximal inhibitory concentration (IC50) data and matched transcriptomic profiles obtained from the Genomics of Drug Sensitivity in Cancer database (18). Subtype-specific drug response predictions were generated using the R package oncoPredict (v0.2). Subtype 1 was classified as chemotherapy-resistant based on significantly higher IC50 values (false discovery rate [FDR]-adjusted P < 0.05).

### 2.8 Gene set enrichment analysis

Gene set enrichment analysis (GSEA) is used to determine whether predefined gene sets exhibit statistically significant differences between biological states. This study assessed differential expression across OC subtypes using limma to calculate log2FC values. GSEA was implemented via clusterProfiler on a ranked gene list ordered by log2FC magnitude. The analyses involved 1,000 gene set permutations. We employed the c2.cp.kegg.v7.5.1.symbols reference gene set from the Molecular Signatures Database (MSigDB) (19) and considered gene sets with nominal P < 0.05 to be significantly enriched.

### 2.9 Immune checkpoints and immune infiltration analysis

In total, 79 immune checkpoints were collected from the literature (20), with 66 expressed in the combined TCGA-OV and GSE63885 dataset. These immune cell–expressed molecules regulate immune activation and prevent excessive immune responses. We compared both groups’ expression of common immune checkpoint genes and assessed immune cell infiltration via single-sample GSEA (ssGSEA), which computes enrichment scores per sample. Immune cell data were obtained from TISIDB (21, 22), and immune infiltration across OC subtypes was visualized using the R package ggplot2 (v3.5.2).

### 2.10 Immune response prediction

We predicted immune therapy responses using the tumor immune dysfunction and exclusion (TIDE) tool (23).

### 2.11 Machine learning for feature selection

We employed support vector machine–recursive feature elimination (SVM-RFE), least absolute shrinkage and selection operator (LASSO) regression via the R package glmnet (v2.25.0), and random forest analysis to identify significant features for model construction. Feature importance was evaluated using mean decrease accuracy (MDA) and mean decrease Gini (MDG) scores. The most significant hub genes were selected for subsequent analyses.

### 2.12 Nomogram construction and validation

We constructed a diagnostic nomogram based on hub gene expression using the R package rms. After calculating risk scores, we evaluated the nomogram’s diagnostic performance using calibration plots and receiver operating characteristic (ROC) curves.

### 2.13 Molecular docking and competing endogenous RNA network construction

Molecular docking of HCQ and SSZ with target proteins was performed using Autodock 1.2.2, and binding energies were visualized using PyMOL. To explore competing endogenous RNA (ceRNA) regulation in OC, we predicted hub gene–targeted microRNAs (miRNAs) and associated long noncoding RNAs (lncRNAs) using the miRTarBase, StarBase v2.0, and miRDB databases, enabling construction of a ceRNA network.

### 2.14 Construction of RNA-binding protein–mRNA and transcription factor–mRNA networks

RNA-binding protein (RBP)–mRNA interactions were analyzed using StarBase (24) based on crosslinking and immunoprecipitation sequencing, degradome sequencing, and RNA–RNA interaction data. The RBP–mRNA network was visualized in Cytoscape. Transcription factor (TF)–mRNA interactions were predicted using the hTFtarget database (25) to construct a TF–mRNA network based on human TF chromatin immunoprecipitation sequencing data.

### 2.15 Drug sensitivity assay

Drug cytotoxicity was assessed in SKOV3 OC cells (Cell Bank, Chinese Academy of Sciences). Stock solutions of HCQ, SSZ, niclosamide (NIC), arsenic trioxide (ATO), and cisplatin (CDDP) were prepared in dimethyl sulfoxide or saline and stored under temperature- and light-controlled conditions. Cells were seeded in 96-well plates, allowed to adhere for 24 h, and treated with optimized drug concentrations: HCQ (0.625–100 μM), SSZ (50 μM–10 mM), NIC (0.25–10 μM), ATO (1–15 μM), and CDDP (2–10 μM). Serial dilutions were performed, with blank (medium only) and untreated controls included for normalization. After 48 h of drug exposure, cell viability was quantified using the Cell Counting Kit-8 (CCK-8) assay and calculated as a percentage as follows: (As − Ab)/(Ac −Ab) × 100, where As is the absorbance of drug-treated wells, Ab is the blank (medium-only) absorbance, and Ac is the absorbance of untreated control wells. Dose–response curves were generated via nonlinear regression to determine half-maximal inhibitory concentration (IC_50_) values. Experiments were performed in quadruplicate to ensure statistical robustness.

The SKOV3 cell line was obtained from the Cell Bank of the Chinese Academy of Sciences (Shanghai, China) and authenticated by the supplier. The compounds were sourced as follows: SSZ (HY-14655), HCQ (HY-W031727), NIC (HY-B0497), and CDDP (HY-17394) from MedChemExpress (Monmouth Junction, NJ, USA); and ATO from Harbin Medical University Pharmaceutical Co., Ltd. (Heilongjiang, China).

### 2.16 Retrospective ascites–serum cytokine analysis

We conducted a single-center retrospective study using deidentified records of 18 patients withhistologically confirmed high-grade serous OC treated at the Department of Obstetrics andGynecology, Peking University People’s Hospital between March 2021 and May 2025. The study adhered to the Declaration of Helsinki and was approved by the Institutional Review Board of Peking University People’s Hospital (No. 2022PHB329-002); written informed consent was obtained from all participants. Ascitic and/or serum cytokine panel results were retrieved from the hospital laboratory information system and electronic medical records, and samples obtained on the same day were considered paired. Eligible patients were those with histopathologically confirmed high-grade serous OC who had at least one clinically performed cytokine panel test with retrievable laboratory reports; intra-abdominal infection or abscess was an exclusion criterion. The routine panel included 14 cytokines (listed in **Supplementary Method 2.16**). Based on the panel results, IL-6, IL-8, and IL-10 showed the most prominent differencesbetween ascites and serum and were therefore selected for the primary analyses. Values reportedbelow the assay reportable range were imputed as one-half of the corresponding reporting threshold (**Supplementary Method 2.16**). The primary comparisons were between ascitic and serum cytokine levels; between-group differences were assessed using Welch’s t-test, and paired t-tests were applied to same-day paired samples. Benjamini–Hochberg correction was applied when comparing cytokine levels across patient groups. All analyses were performed in R (version 4.1.2) with a two-sided α = 0.05.

### 2.17 Statistical analysis

All analyses were conducted in R. Kaplan–Meier curves and log-rank tests were employed to compare survival rates. Data visualization was achieved using ggplot2, and risk scores were calculated using the R package survival. Heatmaps were generated using the R package pheatmap. Statistical significance was assessed through t-tests or analysis of variance for normally distributed variables and Wilcoxon or Kruskal–Wallis tests for non-normal data. P < 0.05 was considered statistically significant, with adjustments made for multiple comparisons as noted for specific analyses.

## 3 Results

### 3.1 Transcriptomic differential analysis

**Figure 2:**
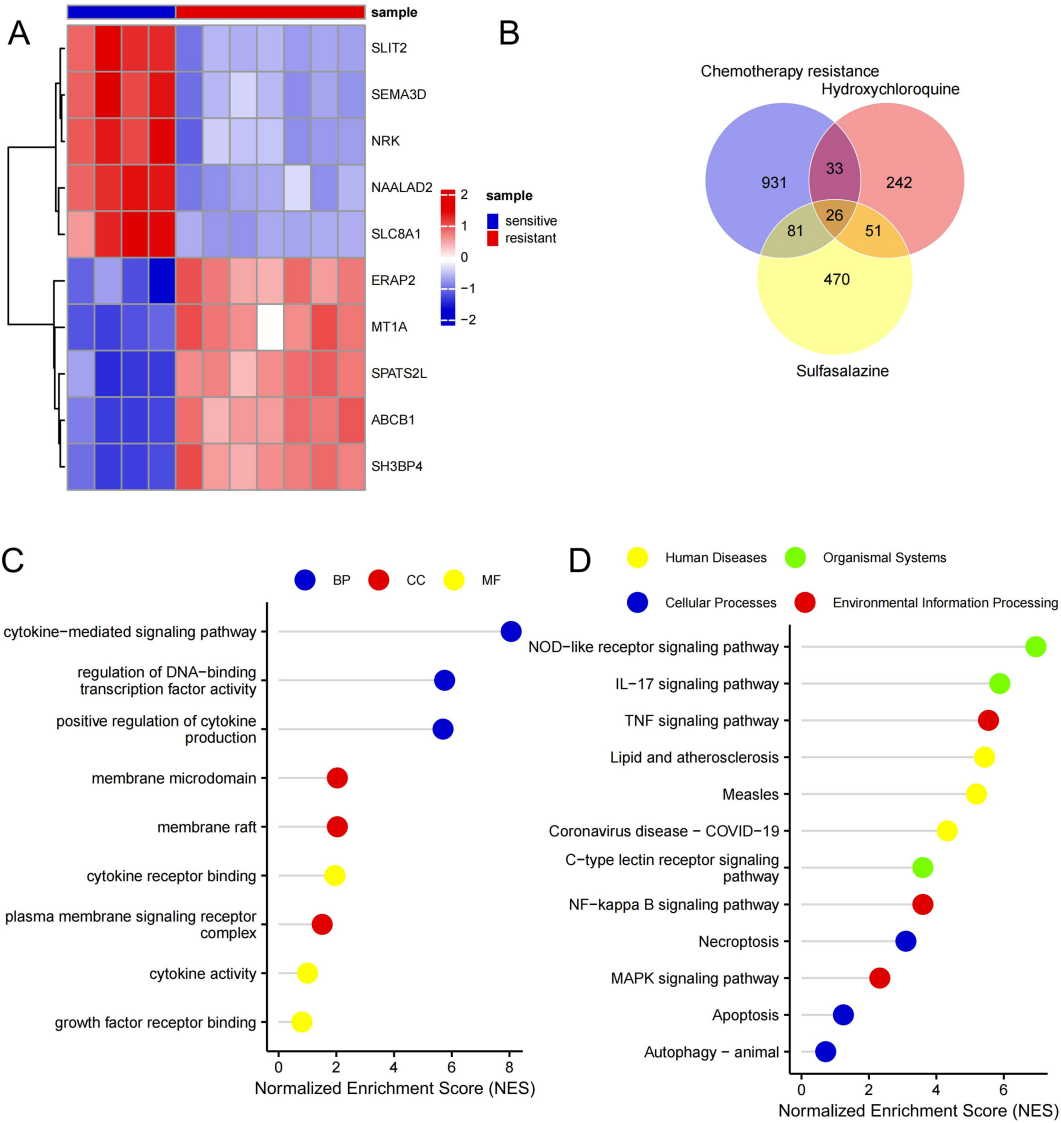
Identification and enrichment analysis of therapeutic targets for hydroxychloroquine–sulfasalazine in reversing paclitaxel–platinum chemoresistance. **(A)** Heatmap showing expression patterns of the top 10 most significant DEGs. **(B)** Venn diagram identifying core therapeutic targets for the hydroxychloroquine–sulfasalazine combination in reversing paclitaxel–platinum chemoresistance. **(C)** GO enrichment analysis of overlapping targets. **(D)** KEGG pathway enrichment of overlapping targets. DEGs, differentially expressed genes; GO, Gene Ontology; KEGG, Kyoto Encyclopedia of Genes and Genomes.

To study genes associated with paclitaxel resistance, we integrated transcriptome datasets GSE230667 (1 paclitaxel-resistant and 1 paclitaxel-sensitive) and GSE73935 (6 paclitaxel-resistant samples and 3 sensitive samples), ultimately obtaining 7 resistant samples and 4 sensitive ones. Differential expression analysis was performed between these two groups, identifying gene clusters related to paclitaxel resistance. Comparative analysis between resistant and sensitive groups revealed 180 DEGs (|log2FC| > 1, FDR-adjusted P < 0.05), encompassing 72 upregulated and 108 downregulated genes in resistant samples. The top five upregulated genes (ABCB1, SH3BP4, SPATS2L, ERAP2, and MT1A) and downregulated genes (SLIT2, NAALAD2, SEMA3D, NRK, and SLC8A1) were further illustrated using heatmaps and box/violin plots (**Figure 2A**).

A Venn diagram of paclitaxel/platinum resistance–associated genes and HCQ/SSZ molecular targets highlighted 26 overlapping genes (**Figure 2B**; **Supplementary Table 1**). All of these genes were expressed in ovarian cancer in the training set, and were therefore considered the major candidate targets through which combined HCQ+SSZ treatment may reverse chemoresistance to paclitaxel plus platinum agents. To explore the potential pathways through which these core targets may function, we performed GO term and KEGG pathway enrichment analyses of the overlapping genes and visualized the results using lollipop plots. GO enrichment indicated that these genes were mainly involved in biological processes (BP) such as the cytokine-mediated signaling pathway (GO:0019221), inhibitor of kappa B (I-κB) phosphorylation (GO:0007252), and regulation of DNA-binding transcription factor activity (GO:0051090); cellular components (CC) including the CD40 receptor complex (GO:0035631), membrane raft (GO:0045121), and membrane microdomain (GO:0098857); and molecular functions (MF) such as cytokine receptor binding (GO:0005126), insulin receptor substrate binding (GO:0043560), and cytokine activity (GO:0005125) (**Figure 2C**). KEGG analysis showed that the overlapping genes were significantly enriched in pathways including the interleukin-17 (IL-17) signaling pathway (hsa04657), NOD-like receptor signaling pathway (hsa04621), and TNF signaling pathway (hsa04668) (**Figure 2D**), suggests that HCQ and SSZ may reverse chemoresistance primarily by attenuating inflammation-driven, NF-κB–centered signaling networks that sustain drug efflux and immune evasion in OC.

### 3.2 Target gene-based subtyping via intersubtype drug sensitivity and GSEA

**Figure 3:**
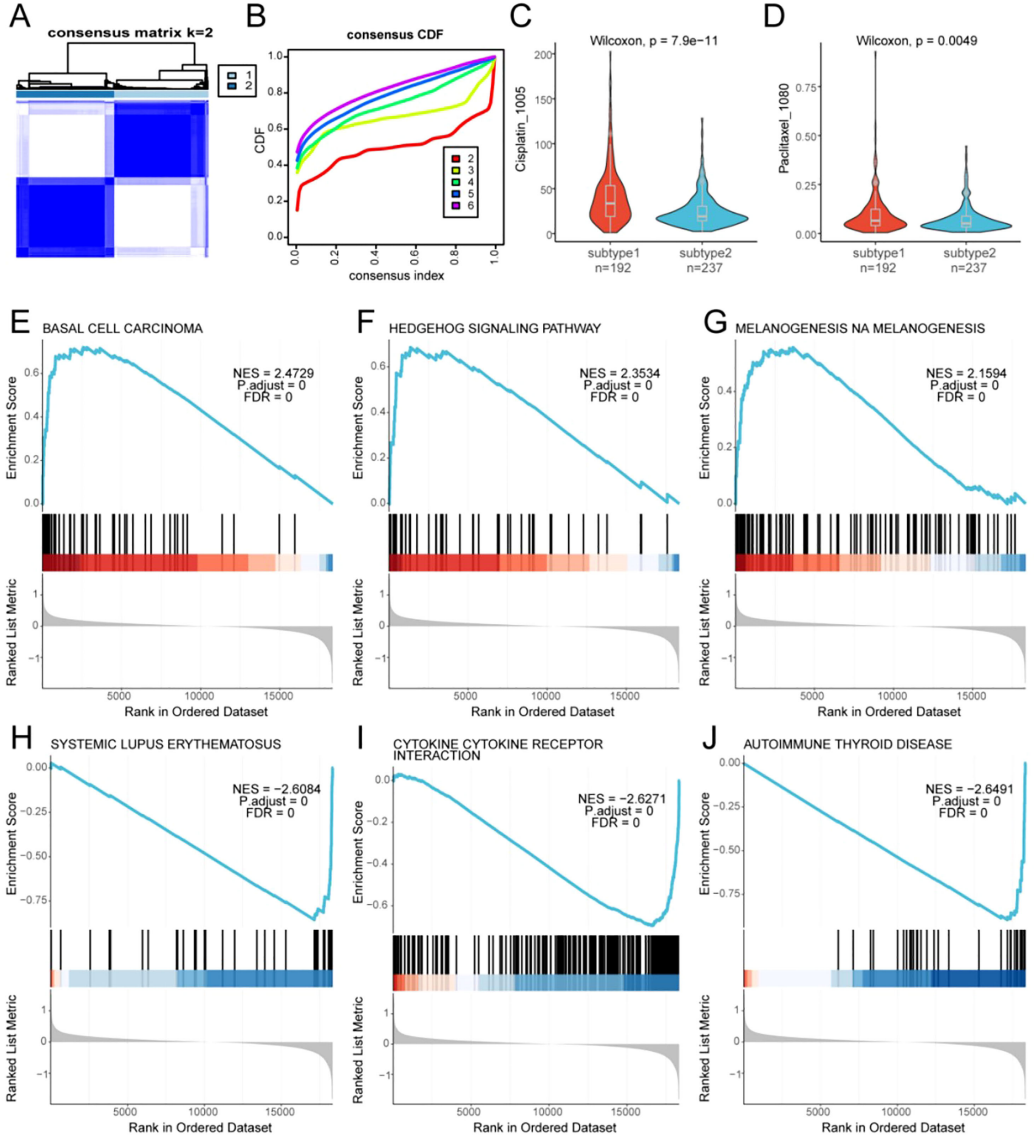
Disease subtyping and intersubtype correlation analysis based on target genes. **(A)** Target gene–based sample subtyping and differential analysis. **(B)** Cumulative distribution function (CDF) curves from consensus clustering. **(C)** Difference in drug sensitivity to Cisplatin_1005 between the subtypes. **(D)** Difference in drug sensitivity to Paclitaxel_1080 between the subtypes. **(E–J)** GSEA results show significant enrichment across subtypes for **(E)** basal cell carcinoma, **(F)** Hedgehog signaling pathway, **(G)** melanogenesis, **(H)** systemic lupus erythematosus, **(I)** cytokine–cytokine receptor interaction, and **(J)** autoimmune thyroid disease. CDF, cumulative distribution function; GSEA, gene set enrichment analysis.

Using the 26 overlapping targets, consensus clustering of 530 OC samples from the integrated TCGA-OV and GSE63885 dataset identified two subtypes: Subtype 1 (n = 239) and Subtype 2 (n = 291), which showed significant differential expression patterns across target genes (**Figures 3A, B**). Chemotherapy sensitivity analysis revealed that Subtype 1 exhibited a reduced response to Cisplatin_1005 (**Figure 3C**) and Paclitaxel_1080 (**Figure 3D**), classifying it as chemoresistant. GSEA (MSigDB, P < 0.05) identified the top enriched pathways, selected via normalized enrichment scores (NES), with FDR = 0 in all cases (**Figures 3E–J** and **Supplementary Table 2**): The chemoresistant. (Subtype1) is significantly enriched in pro-tumorigenic pathways: basal cell carcinoma (NES = 2.47), Hedgehog signaling (NES = 2.35), and melanogenesis (NES = 2.16), among others; at the same time, the sensitive (Subtype 2) is enriched in immune-dysregulation pathways: systemic lupus erythematosus (NES = −2.61), cytokine–cytokine receptor interaction (NES = −2.63), and autoimmune thyroid disease (NES = −2.65) pathways. Consensus clustering stratified OC into a chemoresistant subtype enriched for Hedgehog-driven stemness and redox adaptation programs, and a chemosensitive subtype enriched for immune-regulatory pathways, reinforcing the mechanistic rationale for targeting the inflammatory–survival axis to restore chemosensitivity.

### 3.3 Intersubtype prognostic analysis, immune checkpoint profiling, and TIDE evaluation

**Figure 4:**
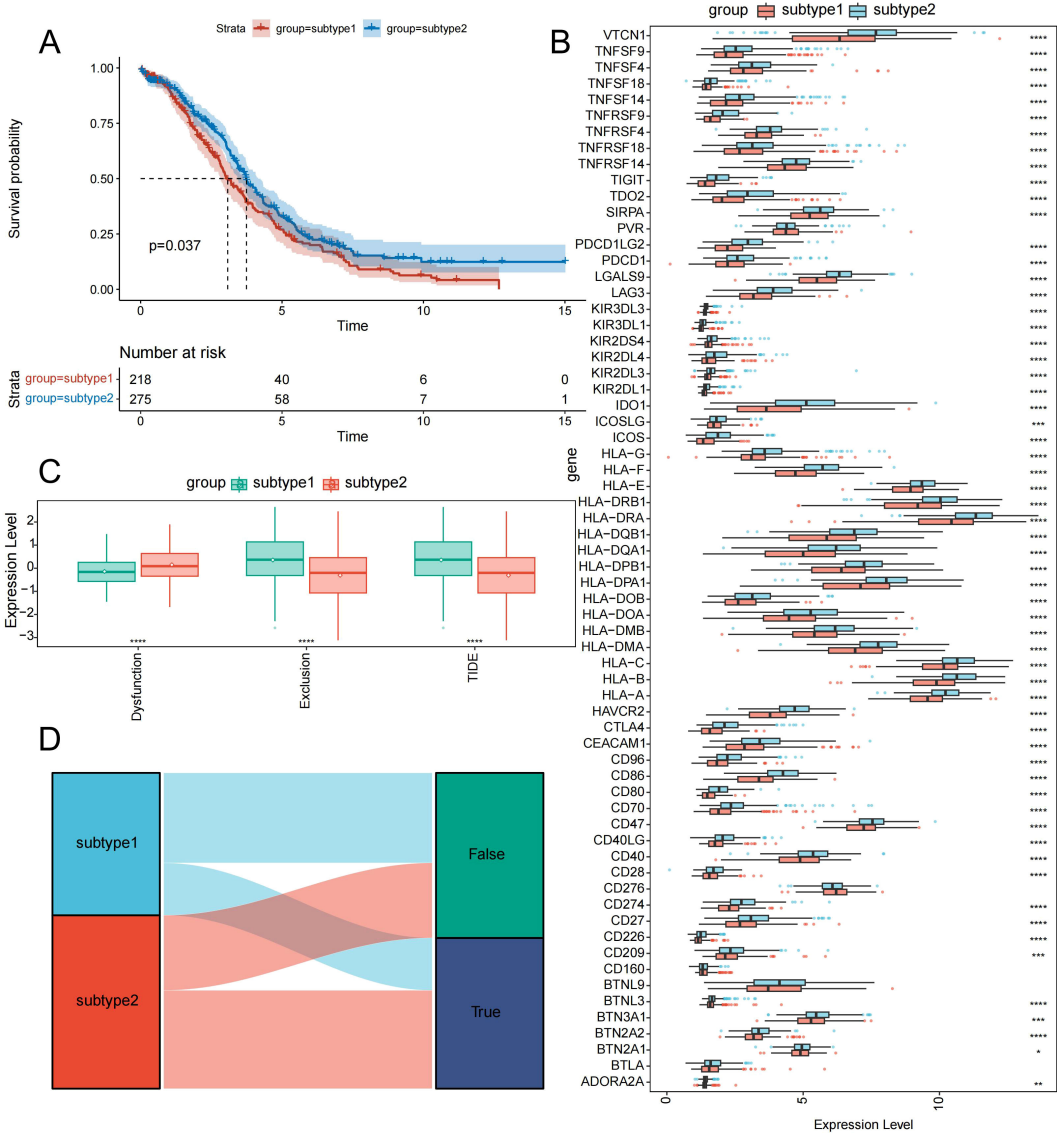
Prognostic, immune checkpoint, and TIDE analyses revealing significant differences between subtypes. **(A)** Kaplan–Meier curves comparing survival between subtypes. **(B)** Boxplot of immune checkpoint gene expression across subtypes. **(C)** Boxplot of TIDE analysis results. **(D)** Sankey diagram of TIDE analysis outcomes, true represents patients who can benefit from immunotherapy, while false represents those who cannot benefit. Statistical significance: ****P < 0.0001, ***P < 0.001, **P < 0.01, and *P < 0.05. TIDE, tumor immune dysfunction and exclusion.

Kaplan–Meier survival analysis demonstrated significant prognostic divergence between subtypes, with chemoresistant Subtype 1 showing markedly poorer outcomes (**Figure 4A**). Immune checkpoint analysis of 62 regulators revealed differential expression across subtypes for 57 genes, with PVR, CD276, CD160, BTNL9, and BTLA showing no intersubtype variation (**Figure 4B**). Further, TIDE analysis showed that Subtype 1 had reduced predicted responsiveness to immune checkpoint blockade (**Figures 4C, D**). The inferior survival outcomes and reduced immune checkpoint blockade responsiveness of chemoresistant Subtype 1, coupled with broad differential expression of immune checkpoint regulators, suggest that resistance is accompanied by deep immunosuppressive remodeling that may limit the efficacy of both conventional and immune-based therapies—further underscoring the need for combination strategies that simultaneously address inflammatory signaling and immune dysfunction.

### 3.4 Immune infiltration profiling

**Figure 5:**
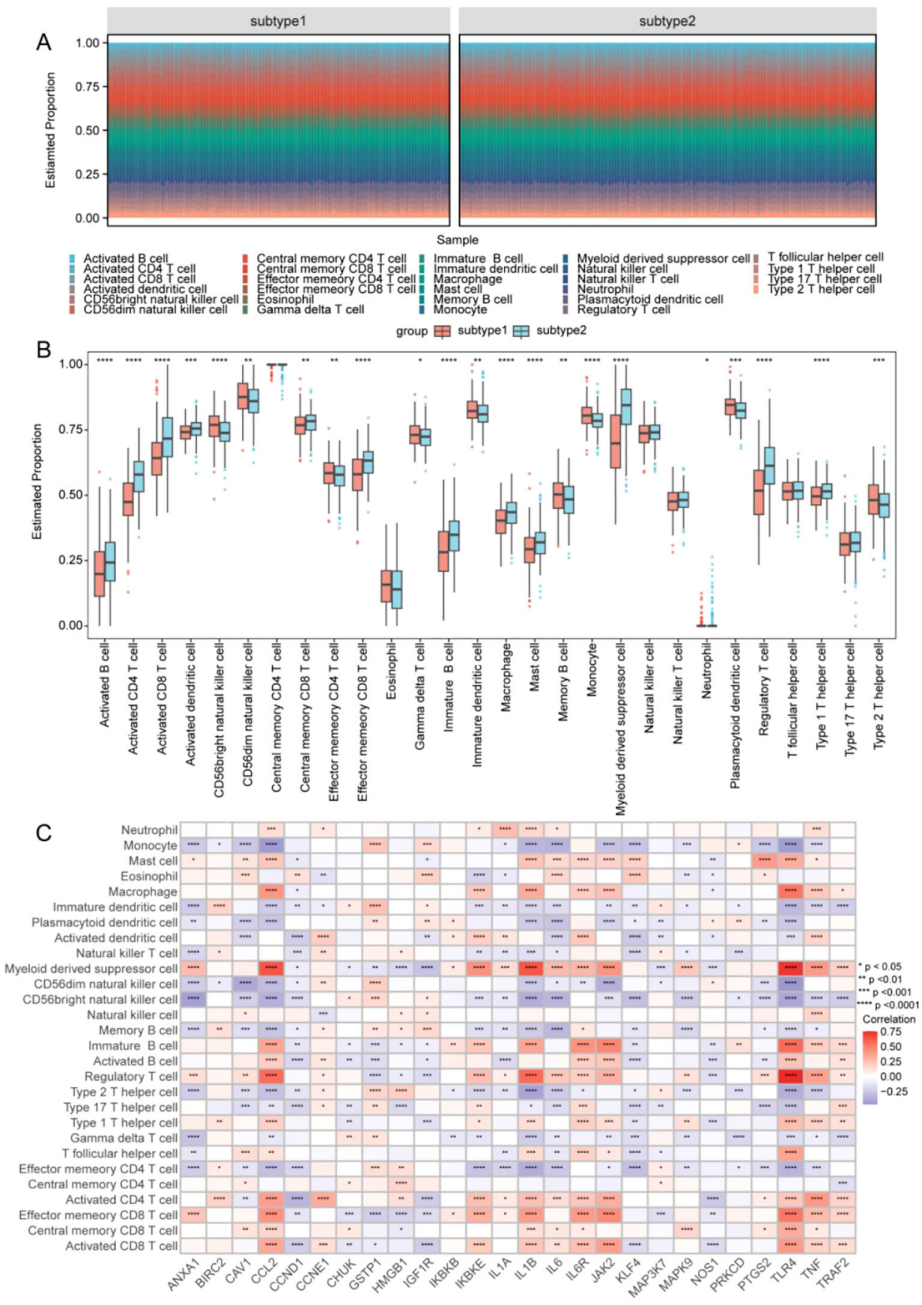
Analysis of immune infiltration between subtypes and correlation analysis with hub genes. **(A)** Stacked bar plot of estimated immune cell population proportions between subtypes. **(B)** Boxplot of subtype-specific immune cell infiltration levels. **(C)** Heatmap of correlations between target genes and immune cell types. Statistical significance: ****P < 0.0001, ***P < 0.001, **P < 0.01, and *P < 0.05.

The infiltration of 28 immune cell types was quantified across OC subgroups using ssGSEA. Relative immune cell composition differed markedly between the two subtypes (**Figure 5A**). In total, 22 cell types exhibited significant differences between subtypes (P < 0.05), whereas no differences were observed in central memory CD4 T cells, eosinophils, natural killer (NK) cells, NK T cells, T follicular helper cells, and type 17 T helper cells (**Figure 5B**). Correlation heatmap analysis revealed predominantly negative associations between target gene expression and immune cell abundance (**Figure 5C**), suggesting potential roles for these genes in shaping immune cell dynamics and clinical prognosis.

### 3.5 Machine learning–based hub gene screening

**Figure 6:**
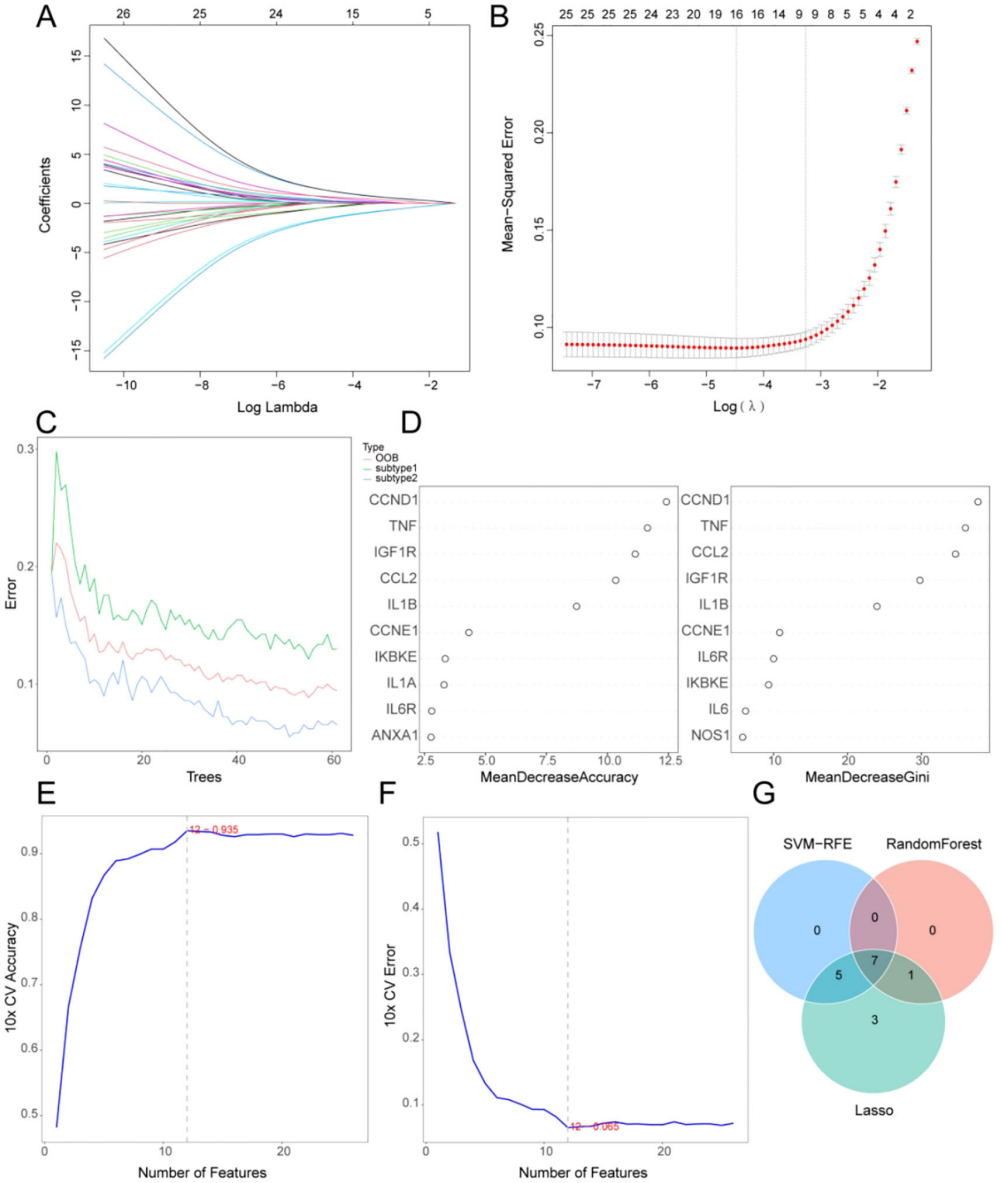
Machine learning–based identification of hub genes in reversing ovarian cancer paclitaxel–platinum chemoresistance. **(A)** Variable coefficient trajectories in LASSO regression. **(B)** Confidence intervals across lambda iterations in LASSO regression. **(C)** Random forest error rate versus classification tree count. **(D)** Top 10 genes ranked by MDA and MDG metrics in random forest analysis. **(E)** SVM-RFE accuracy curve for feature elimination. **(F)** SVM-RFE error rate during feature elimination. **(G)** Venn diagram showing intersecting hub genes across three machine-learning approaches. LASSO, least absolute shrinkage and selection operator; MDA, mean decrease accuracy; MDG, mean decrease Gini; SVM-RFE, support vector machine–recursive feature elimination.

We applied three machine-learning models, namely, LASSO, random forest, and SVM–RFE in the combined TCGA-OV and GSE63885 cohort (N = 530) to identify more critical targets from the current 26 genes. LASSO identified 16 candidate genes (**Figures 6A, B**). Random forest analysis yielded 10 genes based on MDA and MDG metrics, which were refined to 8 nonredundant candidates (**Figures 6C, D**). Additionally, SVM–RFE identified 12 genes through iterative feature elimination (**Figures 6E, F**). Finally, we integrated the three methods, which revealed seven common hub genes that were used for downstream analyses (**Figure 6G**).

### 3.6 Construction and validation of the diagnostic nomogram

**Figure 7:**
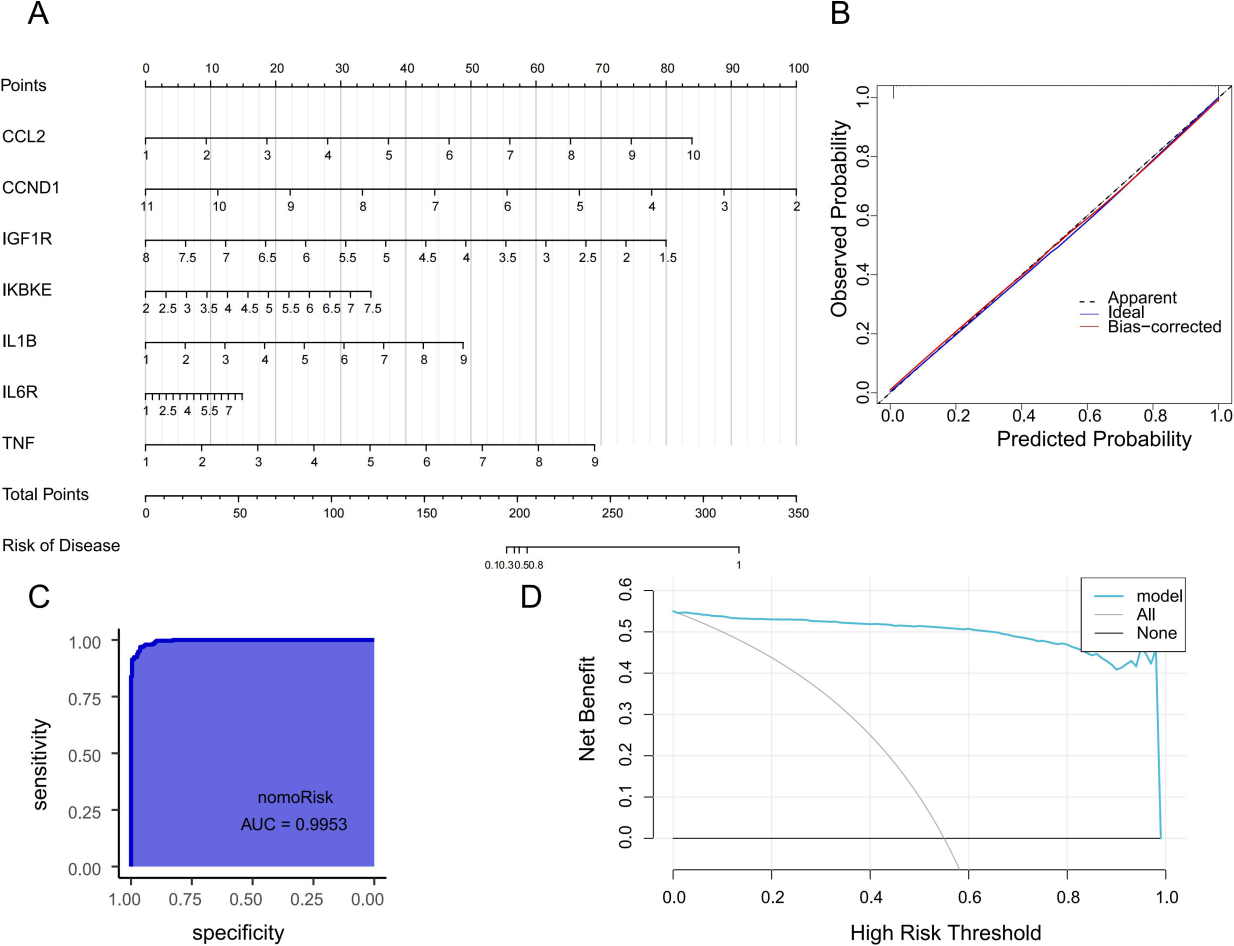
Establishment and validation of a diagnostic model for paclitaxel–platinum chemoresistant ovarian cancer patients. **(A)** Diagnostic nomogram for chemoresistant populations incorporating seven hub genes: CCL2, CCND1, IGF1R, IKBKE, IL1B, IL6R, and TNF. **(B)** Calibration curve indicating the predictive accuracy of the nomogram. **(C)** ROC curve evaluating the nomogram’s clinical utility. ROC: receiver operating characteristic. **(D)** DCA curve of the diagnostic nomogram.

We developed a diagnostic nomogram incorporating the seven hub genes CCL2, CCND1, IGF1R, IKBKE, IL1B, IL6R, and TNF to predict platinum–paclitaxel treatment response and potential clinical benefits from HCQ/SSZ therapy (**Figure 7A**). Calibration curves showed close concordance between predicted and observed probabilities, indicating strong model accuracy (**Figure 7B**), and ROC curve analysis confirmed the robustness of the nomogram’s robust discriminatory performance for identifying chemoresistant populations (area under the curve (AUC) = 0.9953, **Figure 7C**). The diagnostic nomogram was evaluated using decision curve analysis(DCA), which confirmed its favorable clinical applicability (**Figure 7D**).

### 3.7 Molecular docking analysis

**Figure 8:**
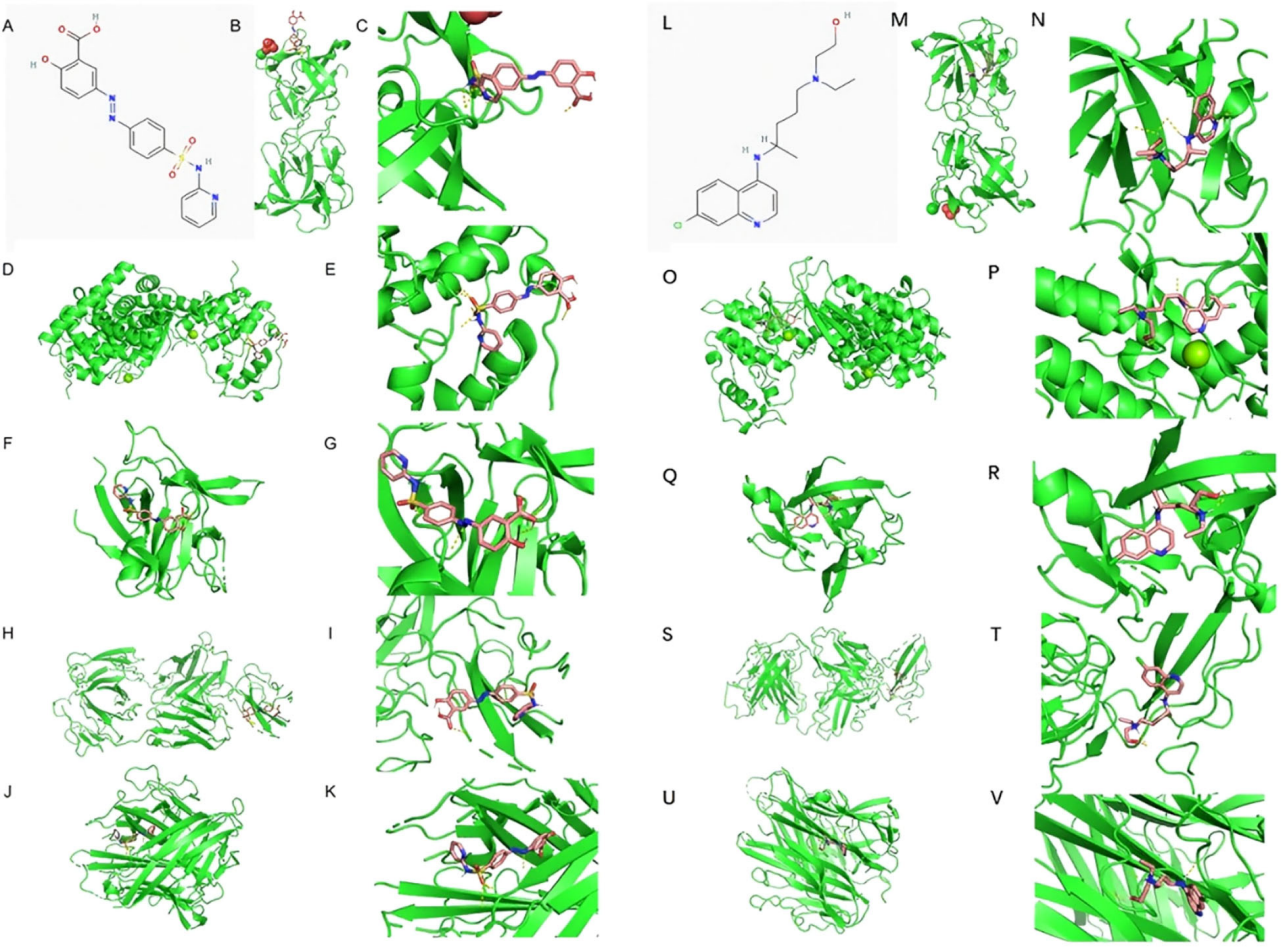
Molecular docking of sulfasalazine and hydroxychloroquine with target proteins. **(A)** Two-dimensional structural representation of sulfasalazine. **(B)** Docking pose of sulfasalazine with CCL2, with **(C)** the binding site magnified. **(D)** Docking pose of sulfasalazine with CCND1, with **(E)** the binding site magnified. **(F)** Docking pose of sulfasalazine with IL1B, with **(G)** the binding site magnified. **(H)** Docking pose of sulfasalazine with IL6R, with **(I)** the binding site magnified. **(J)** Docking pose of sulfasalazine with TNF, with **(K)** the binding site magnified. **(L)** Two-dimensional structural representation of hydroxychloroquine. **(M)** Docking pose of hydroxychloroquine with CCL2, with **(N)** the binding site magnified. **(O)** Docking pose of hydroxychloroquine with CCND1, with **(P)** the binding site magnified. **(Q)** Docking pose of hydroxychloroquine with IL1B, with **(R)** the binding site magnified. **(S)** Docking pose of hydroxychloroquine with IL6R, with **(T)** the binding site magnified. **(U)** Docking pose of hydroxychloroquine with TNF, with **(V)** the binding site magnified.

We assessed the binding potential of HCQ and SSZ to five hub protein targets (CCL2, CCND1, IL1B, IL6R, and TNF) using Protein Data Bank (PDB) database–retrieved structures. Ten ligand–receptor complexes were generated (**Table 1**), with all complexes demonstrating favorable, stable interactions (binding energies: −3.76 to −4.84 kcal/mol for HCQ; −4.08 to −6.99 kcal/mol for SSZ) consistent with therapeutic relevance. Structural docking visualizations highlighted key binding residues and interaction geometries (**Figures 8A–V**), supporting the potential of both compounds to engage chemoresistance-associated targets with high molecular compatibility.

**Table 1:** Docking results for sulfasalazine and hydroxychloroquine with predicted protein targets.

Drug	Target	PDB ID	Binding energy (kcal/Mol)
Sulfasalazine	CCL2	4USP	−4.08
Sulfasalazine	CCND1	6P8E	−4.51
Sulfasalazine	IL1B	8RYS	−6.56
Sulfasalazine	IL6R	8J6F	−4.10
Sulfasalazine	TNF	6OOY	−6.99
Hydroxychloroquine	CCL2	4USP	−4.21
Hydroxychloroquine	CCND1	6P8E	−3.76
Hydroxychloroquine	IL1B	8RYS	−4.76
Hydroxychloroquine	IL6R	8J6F	−3.87
Hydroxychloroquine	TNF	6OOY	−4.84

### 3.8 Regulatory network analysis based on hub genes

**Figure 9:**
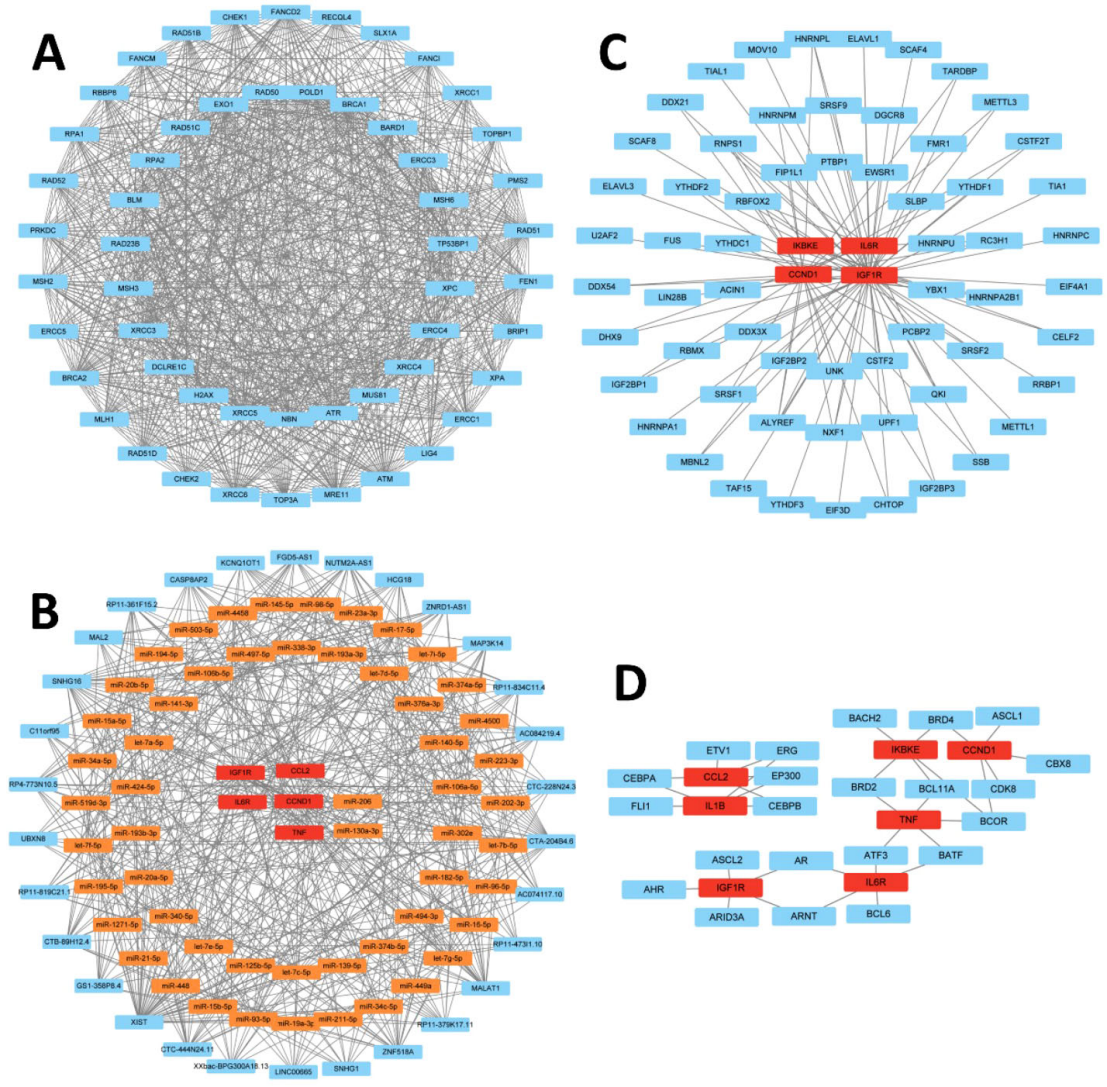
Regulatory network analyses based on hub genes. **(A)** Protein–protein interaction network linking hub genes with chemoresistance-associated genes. **(B)** Hub gene–centered lncRNA–miRNA–mRNA ceRNA network (blue: lncRNAs; yellow: miRNAs; red mRNAs.) **(C)** RBP–mRNA regulatory network (blue: RBPs; red: mRNAs). **(D)** TF–mRNA regulatory network (blue: TFs; red: mRNAs). RBP, RNA-binding protein; lncRNA, long noncoding RNA; miRNA, microRNA; ceRNA, competing endogenous RNA; TF, transcription factor.

To map interactions among the seven hub (**Supplementary Table 3**) and chemoresistance-associated genes (**Supplementary Tables 4-5**), we constructed a high-confidence (score ≥ 0.7) protein–protein interaction network using Search Tool for the Retrieval of Interacting Genes/Proteins (STRING). Cytoscape visualization revealed 925 interacting proteins. Molecular Complex Detection (MCODE) clustering (default k-means clustering settings) identified multiple subnetworks, including a top-scoring module (**Figure 9A**; **Supplementary Table 6**) and hub gene–enriched protein clusters xxx(**Supplementary Figures 2–4**; **Supplementary Tables 7–9**).

To characterize the regulatory mechanisms of the seven hub genes in OC, we conducted multidimensional network analyses of ceRNA, RBP, and TF. The ceRNA network identified IGF1R, CCL2, IL6R, CCND1, and TNF as core nodes, which formed an interaction network comprising 30 lncRNAs, 55 miRNAs, and 5 mRNAs (interaction degree ≥10; **Figure 9B**; **Supplementary Table 10**). Meanwhile, StarBase-derived RBP–mRNA mapping for IKBKE, IL6R, CCND1, and IGF1R produced a 65-node regulatory network (61 RBPs and 4 mRNAs) with 102 regulatory edges (**Figure 9C**; **Supplementary Table 11**). Additionally, TF–mRNA interactions extracted from hTFtarget identified 22 TFs interacting with the hub genes (**Figure 9D**; **Supplementary Tables 12**, **S13**). Together, these multilayered networks define the regulatory landscape supporting chemoresistance in OC and highlight potential therapeutic intervention points.

### 3.9 Drug sensitivity testing, ascites–serum cytokine analysis, and clinical outcomes

**Figure 10:**
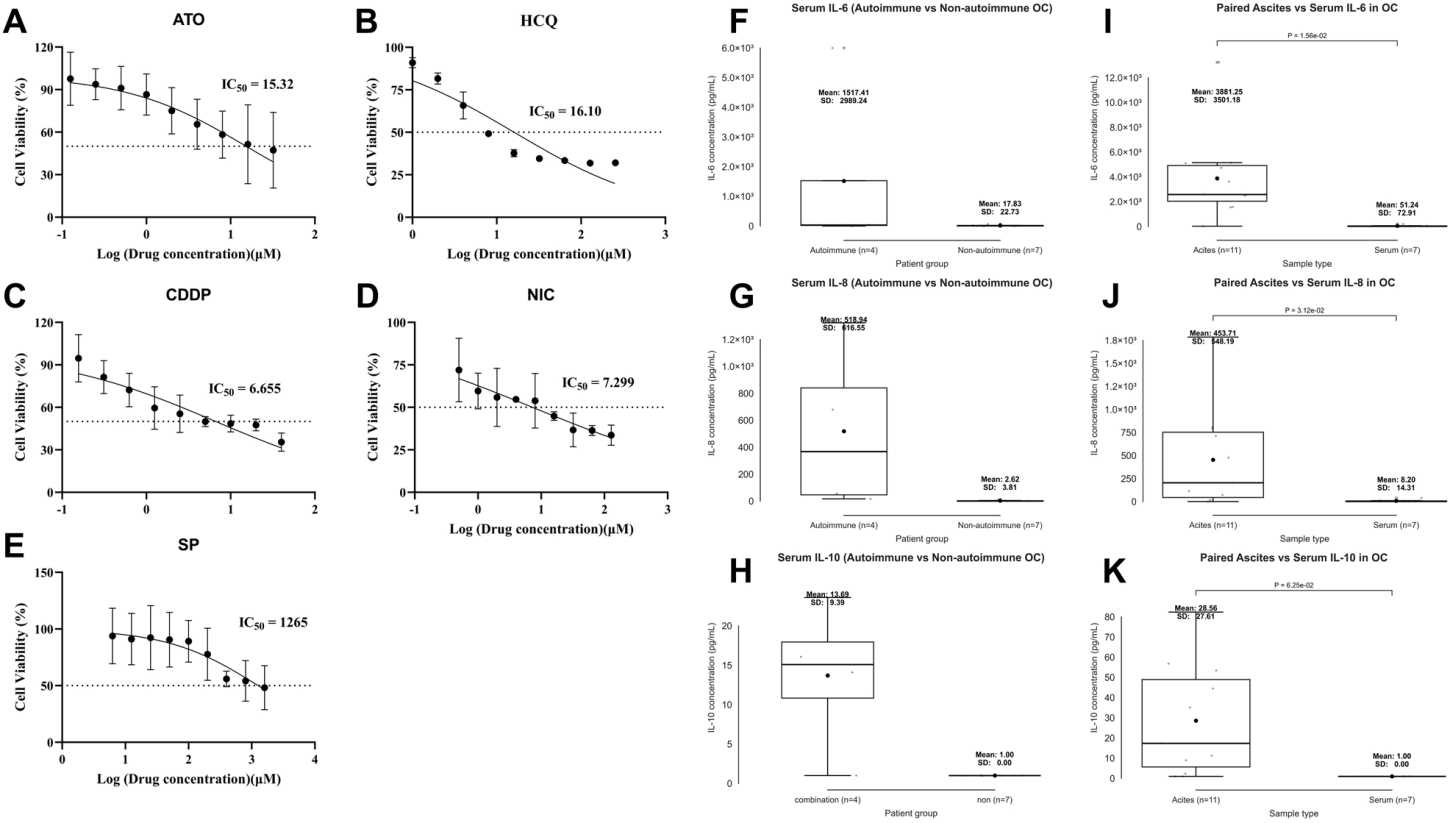
Integrated pharmacologic and cytokine profiling in ovarian cancer. **(A–E)** Dose–response curves for **(A)** arsenic trioxide (ATO; PML-RARα degrader), **(B)** hydroxychloroquine (HCQ; autophagy inhibitor), **(C)** cisplatin (CDDP; DNA crosslinker), **(D)** niclosamide (NIC; Signal transducer and activator of transcription 3 (STAT3)/Wnt signaling inhibitor), and **(E)** sulfasalazine (SSZ; NF-κB/glutathione inhibitor); all agents reduced cell viability in a dose-dependent manner. IC_50_ values are log-transformed (μM). **(F–H)** Retrospective serum cytokine profiling revealed higher IL-6, IL-8, and IL-10 levels in HGSOC patients with autoimmune disease than in those without, shown for **(F)** IL-6, **(G)** IL-8, and **(H)** IL-10. **(I–K)** In patients without autoimmune disease, ascites cytokine concentrations were higher than matched serum levels, shown for **(I)** IL-6, **(J)** IL-8, and **(K)** IL-10.All boxplots show medians, interquartile ranges, and individual data points. Welch’s t-tests were used for between-group comparisons.

To assess the cytotoxic effects, full dose–response curves were generated for five agents (**Figures 10A–E**), and IC_50_ values were quantified in OC cell models and log_10_-transformed for analysis. ATO showed an IC_50_ of 15.32 μM (**Figure 10A**), HCQ 16.10 μM (**Figure 10B**), CDDP 6.655 μM (**Figure 10C**), NIC 7.299 μM (**Figure 10D**), and SSZ 1,265 μM (**Figure 10E**). Among these compounds, CDDP exhibited the greatest cytotoxicity, and all agents reduced cell viability, indicating antitumor activity across mechanistically distinct compounds.

Retrospective serum cytokine profiling revealed markedly elevated IL-6, IL-8, and interleukin-10 (IL-10) levels in patients with high-grade serous ovarian cancer (HGSOC) and coexisting autoimmune disease (n = 4; 1 with rheumatoid arthritis and 3 with tumor-associated dermatomyositis) compared with those without autoimmune disease (n = 7), although no statistical comparisons were performed due to unequal sample sizes. The mean ± SD values (autoimmune vs. nonautoimmune HGSOC groups) were as follows: IL-6, 1,517.41 ± 2,989.24 vs. 17.83 ± 22.73 pg/mL; IL-8, 518.94 ± 616.55 vs. 2.62 ± 3.81 pg/mL; and IL-10, 13.69 ± 9.39 vs. 1.00 ± 0.00 pg/mL (**Figures 10F–H**).

In prospective analyses of patients without autoimmune disease, cytokine expression levels were significantly higher in ascitic fluid than in matched serum samples: IL-6 (ascites vs. serum), 3,881.25 ± 3,501.18 vs. 51.24 ± 72.91 pg/mL (P = 1.56 × 10^−2^; **Figure 10I**); IL-8, 453.71 ± 548.19 vs. 8.20 ± 14.31 pg/mL (P = 3.12 × 10^−2^; **Figure 10J**); IL-10, 28.56 ± 27.61 vs. 1.00 ± 0.00 pg/mL (P = 6.25 × 10^−2^; **Figure 10K**). Welch’s t-tests were used for all paired comparisons.

These results reveal a consistent inflammatory signature in patients with OC: serum cytokine levels were elevated in patients with autoimmune comorbidities, and the same cytokines are markedly enriched in ascites across patients (without autoimmune diseases), indicating a pronounced local inflammatory response within the tumor microenvironment. These results highlight the convergence of systemic and local immune activation in OC, providing a mechanistic rationale for the sensitivity to immunomodulatory agents such as HCQ and SSZ.

Among the autoimmune disease–complicated HGSOC cases with cytokine data, three patients treated in the long term with HCQ and/or SSZ showed significantly prolonged overall survival: 126 months, alive at last follow-up; 114 months, deceased due to chemotherapy-induced myelosuppression; 26 months, alive with no recurrence (initially International Federation of Gynecology and Obstetrics (FIGO) stage IV and treatment-sensitive). In contrast, the untreated patient survived for 20 months. All patients were diagnosed at advanced FIGO stages (III–IV). A retrospective analysis of over 40 cases of ovarian cancer patients with autoimmune diseases who achieved long-term survival through prolonged HCQ and/or SSZ therapy will be presented in a separate manuscript.

## 4 Discussion

OC accounts for ~206,839 global deaths worldwide each year, and >80% of patients with advanced disease develop chemoresistant recurrence following surgery and chemotherapy (1, 26). Platinum-resistant disease affects ~25% of patients and is associated with a median survival <12 months (27), driven by NF–κB/IL–17/TNF–mediated inflammation, ABCB1 upregulation, and NLR family pyrin domain containing 3 (NLRP3) inflammasome activation (28–31). Current therapeutic strategies remain insufficient to adequately counter this microenvironment-driven resistance (32). Notably, patients with OC and comorbid rheumatic disorders receiving continuous antirheumatic therapy have demonstrated improved oncologic outcomes (3), suggesting that autoimmunity and cancer share targetable inflammatory mechanisms. As such, repurposing antirheumatic drugs—particularly HCQ, which sensitizes cancer cells to cytotoxic agents via TLR9/NF-κB modulation and macrophage reprogramming (4, 5), and SSZ, which reverses drug resistance through ferroptosis induction (6, 8)—to counter chemoresistance represents a mechanistically rational therapeutic strategy. Using platinum resistance–associated transcriptomic datasets alongside HCQ/SSZ target databases, we identified 26 consensus genes at the intersection of chemoresistance, drug targets, and OC progression that may mediate the restoration of chemosensitivity by these agents.

Gene enrichment analyses implicated cytokine signaling (IL-17/TNF), NF-κB activation (via IκB phosphorylation and ABCB1 upregulation), and NOD-like receptor pathways (e.g., NLRP3-driven IL-1β release) as core drivers sustaining inflammation and drug efflux in chemoresistant OC. Notably, these pathways—and the key genes identified (IL6R, TNF, NLRP3, and ABCB1)—also underlie autoimmune pathogenesis, reflecting shared inflammatory mechanisms (31, 33). In autoimmune disorders, dysregulated IL-17/TNF signaling and NF-κB hyperactivation promote tissue injury (34); in cancer, these same pathways sustain tumor-associated inflammation and drive drug resistance through antiapoptotic protein upregulation (e.g., BCL-2) and ABCB1-mediated drug efflux (31, 35). HCQ and SSZ converge on these pathways through complementary mechanisms: HCQ disrupts membrane raft–dependent ABCB1 function and inhibits TLR/autophagy and interferon signaling (36), whereas SSZ suppresses IKBKE-mediated NF-κB activation and ferroptosis via dihydroorotate dehydrogenase inhibition (37–40). Their combined efficacy likely reflects coordinated targeting of conserved stress-response networks that sustain chronic inflammation, supporting their potential as cost-effective strategies for overcoming microenvironment-mediated chemoresistance (41, 42).

The seven machine learning–identified hub genes (CCL2, CCND1, IGF1R, IKBKE, IL1B, IL6R, and TNF) are expressed and have been reported to be upregulated in ovarian cancer (43–49). The seven hub genes converge on two core oncogenic axes—NF-κB–driven inflammation and PI3K/AKT–mediated survival—that collectively sustain chemoresistance by coupling immune evasion with antiapoptotic signaling (43, 46, 50). Within the inflammatory axis, IKBKE, IL1B, IL6R, and TNF form a self-reinforcing network: IKBKE amplifies NF-κB activity to upregulate BCL-2 and programmed death-ligand 1 (PD-L1) (51), while IL1B/IL6R–STAT3 co-activation sustains T-cell exhaustion and CCL2–CCR2–mediated recruitment of tumor-associated macrophages (TAMs) and myeloid-derived suppressor cells (MDSCs) (52–54), ultimately entrenching immune exclusion. In parallel, IGF1R–PI3K/AKT signaling maintains stemness, and CCND1-driven cell cycle acceleration enables escape from paclitaxel-induced mitotic arrest (46, 50). HCQ and SSZ target this architecture at complementary nodes: HCQ dismantles TLR/IL-6/STAT3 signaling and repolarizes TAMs toward an M1 phenotype (54), whereas SSZ suppresses IKBKE-mediated NF-κB activation and induces ferroptosis (11, 33, 34). Their combined action thus addresses both the inflammatory and survival components of hub-driven resistance, offering a mechanistically coherent strategy to restore drug sensitivity and immune competence in refractory OC.

Within this framework, HCQ and SSZ are notable for their well-defined anti-inflammatory profiles. HCQ reduces the production of multiple proinflammatory cytokines (e.g., IL-1, IL-2, IL-6, and IL-17) and inhibits endosomal TLR7/9 signaling to suppress innate immune activation (55, 56). Meanwhile, SSZ blocks NF-κB activation by inhibiting IκB kinase (IKK)-dependent inhibitor of kappa B alpha (IκBα) phosphorylation and degradation, reducing downstream inflammatory gene expression (including that of IL-1β and IL-6) (38). These mechanisms may thus contribute to chemosensitization during active therapy (by mitigating cytokine-driven survival and efflux programs) and recurrence suppression during maintenance (by attenuating chronic inflammatory and immunosuppressive cues). Our pharmacological validation, network analyses, and clinical observations reinforce this rationale and support further translational development of HCQ/SSZ as chemotherapy adjuncts and contributors to maintenance strategies in OC.

The identification of two molecularly distinct OC subtypes with divergent chemotherapy sensitivity underscores the heterogeneity of resistance mechanisms. In chemoresistant Subtype 1, enrichment of Hedgehog signaling and basal cell carcinoma pathways reflects activation of GLI-linked developmental programs associated with tumor cell plasticity, stem-like features, and therapy tolerance (57–59); concomitant melanogenesis enrichment may indicate metabolic and redox rewiring that buffers oxidative stress and enhances cytotoxic resistance (60, 61). In contrast, the immune-related pathway enrichment in chemosensitive Subtype 2—spanning cytokine–cytokine receptor interaction, systemic lupus erythematosus, and autoimmune thyroid disease—suggests a more immune-engaged microenvironment favorable to chemotherapy-induced immunogenic cell death (62, 63). Notably, several of the 26 overlapping targets (e.g., TNF, IL6R, IL1B, and IKBKE) converge on cytokine/NF-κB signaling nodes directly modulated by HCQ and SSZ, supporting the hypothesis that combined therapy may enhance platinum–taxane sensitivity by attenuating Hedgehog-driven survival programs and dampening NF-κB–coupled inflammatory signaling.

The marked suppression of cytokine–cytokine receptor interactions in chemoresistant tumors further underscores the dual function of inflammatory signaling. Although chronic TNF/IL-17 activity in Subtype 1 promotes NF-κB-driven survival and ABCB1-mediated drug efflux, HCQ and SSZ may counter this process by restoring apoptotic sensitivity through pathway inhibition (33, 35, 64, 65). Immune profiling identified differential expression of 57 out of 62 immune checkpoints, suggesting variable immunotherapy responsiveness. Subtype 2 showed elevated expression of immune checkpoint genes, indicating greater immunotherapy benefit. TIDE analysis further corroborated these observations. This subtyping-based framework supports early identification of high-risk patients and enables more tailored therapeutic planning.

Furthermore, immune profiling revealed significant variation in 22 immune cell populations between subtypes, emphasizing the impact of immune microenvironment remodeling on chemoresistance. These findings indicate a strong correlation between drug resistance and immune infiltration. TAMs, predominantly M2-polarized, suppress CD8+ T-cell activity and recruit regulatory T cells (Tregs) via IL-10 and transforming growth factor beta (TGF-β), maintaining an immunosuppressive niche (35, 66), thereby exacerbating chemoresistance (67). Treg infiltration predicts poor prognosis (68), synergistically enhancing immune evasion (69). MDSCs expand after chemotherapy and impair T-cell function through reactive oxygen species production, arginine depletion, and IL-10 secretion, directly contributing to platinum resistance (70, 71). Exhausted tumor-infiltrating lymphocytes (TILs) express PD-1 and T-cell immunoglobulin and mucin-domain containing-3 (TIM-3) while displaying impaired effector function, whereas tumor cells upregulate PD-L1 via NF-κB signaling to suppress antitumor immunity (72, 73). Based on these findings, we hypothesize that HCQ/SSZ therapy may overcome chemoresistance in OC by targeting immunosuppressive cells (TAMs, Tregs, and MDSCs) while restoring effector immunity (TILs and NK cells).

To validate these findings, we integrated *in vitro* pharmacological assays, clinical outcome analyses, and ascites cytokine profiling. Elevated IL-6, IL-8, and IL-10 levels in OC ascites confirmed a proinflammatory and immunosuppressive tumor microenvironment consistent with the hub-driven resistance architecture described above. *In vitro*, HCQ and SSZ inhibited OC cell growth, corroborating their direct antitumor activity beyond immunomodulation. Clinically, patients with OC and autoimmune comorbidities receiving long-term HCQ and/or SSZ therapy achieved markedly prolonged survival—in some cases exceeding 10 years—providing the initial translational rationale for this study. Collectively, these converging lines of evidence support HCQ and SSZ as cost-effective adjuncts or maintenance therapeutics with measurable prognostic impact, warranting broader integration into OC management.

This study has several limitations. First, molecular docking and transcriptomic analyses were based on in silico predictions and publicly available datasets, which may introduce bias and require experimental validation. Second, *in vitro* drug sensitivity assays were performed in a limited number of cell models, and the findings may not fully generalize across the heterogeneity of OC subtypes. Third, because paired clinical specimens before and after HCQ/SSZ treatment were not prospectively collected, we did not perform treatment-linked sequencing analyses, which limited our ability to further interrogate the functional relevance of the predicted targets in patient-derived settings. Finally, the cytokine profiling cohort was small, and retrospective serum/ascites analyses lacked longitudinal immune monitoring. These limitations highlight the need for validation in multi-lineage models, controlled *in vivo* studies, and prospective clinical investigations; ongoing and future work will incorporate systematic pre-/post-treatment biospecimen collection and multi-omics profiling to address these gaps.

## 5 Conclusions

This study establishes the combination of HCQ and SSZ as a promising therapy for overcoming platinum–taxane resistance in OC by targeting the inflammatory–immune microenvironment. Integrated multiomics analyses identified seven hub genes (CCL2, CCND1, IGF1R, IKBKE, IL1B, IL6R, and TNF) converging on core resistance mechanisms; that is, sustained inflammation, survival signaling, and immune suppression. Molecular docking confirmed potential HCQ/SSZ binding to key targets, supporting their roles in disrupting NF-κB, STAT3, and related pathways. A diagnostic model based on these hub genes enables early detection of chemoresistance and supports patient stratification. This study, motivated by clinical observations followed by confirmatory analyses, included network pharmacology validation consistent with observed clinical outcome. Building on this foundation, we advance the exploration of repurposed antirheumatic drugs as chemosensitizers and maintenance agents in OC. By linking clinical observations with mechanistic evidence, we aim to establish a therapeutic paradigm in which HCQ and SSZ, combined with standard chemotherapy, reverse resistance; enhance drug response; and promote durable remission, delayed recurrence, or sustained nonrecurrence. Moreover, our findings may inform long-term management strategies for initial OC treatment, offering a clinically translatable approach to reduce relapse and improve survival.

## Data Availability

The datasets analyzed in this study are publicly available. Gene expression data were obtained from the Gene Expression Omnibus (GEO, https://www.ncbi.nlm.nih.gov/geo/) under accession numbers GSE230667, GSE73935, and GSE63885. Transcriptomic and clinical data for ovarian serous cystadenocarcinoma were obtained from The Cancer Genome Atlas (TCGA-OV) and are publicly available through the Genomic Data Commons (GDC, https://gdc.cancer.gov/). All data relevant to this study are included in the article and/or its **Supplementary Material**.

## Supplementary material

Supplementary Figure 1 PCA plots before and after data merging. PCA plots of TCGA-OV and GSE63885 before **(A)** and after **(B)** merging.

Supplementary Figure 2–4 display the six hub gene–enriched protein clusters centered on IGF1R, IL1B, CCND1, IKBKE, TNF, and CCL2, respectively.

## Glossary

ATO: Arsenic trioxide
ATO: Arsenic trioxide
AUC: Area under the curve
BP: Biological process
CC: Cellular component
CCK-8: Cell Counting Kit-8
CDDP: Cisplatin
ceRNA: Competing endogenous RNA
DCA: Decision curve analysis
DEG: Differentially expressed genes
DMSO: Dimethyl sulfoxide
FDR: False discovery rate
FIGO: International Federation of Gynecology and Obstetrics
GDSC: Genomics of Drug Sensitivity in Cancer
GEO: Gene Expression Omnibus
GO: Gene Ontology
GSEA: Gene set enrichment analysis
HCQ: Hydroxychloroquine
HGSOC: High-grade serous ovarian cancer
IC50: Half-maximal inhibitory concentration
IκB: Inhibitor of kappa B
IKK: IκB kinase
IL: Interleukin
IRB: Institutional Review Board
JAK: Janus kinase
KEGG: Kyoto Encyclopedia of Genes and Genomes
KM: Kaplan–Meier
LASSO: Least absolute shrinkage and selection operator
lncRNA: Long noncoding RNA
LOD: Limit of detection
log2FC: Log2 fold change
MDA: Mean decrease accuracy
MDG: Mean decrease Gini
MCODE: Molecular Complex Detection
MDSC: Myeloid-derived suppressor cell
MF: Molecular function
miRNA: MicroRNA
MSigDB: Molecular Signatures Database
NES: Normalized enrichment score
NF-κB: Nuclear factor kappa B
NIC: Niclosamide
NK: Natural killer
NOD: Nucleotide-binding oligomerization domain
OC: Ovarian cancer
PCA: Principal component analysis
PDB: Protein Data Bank
PD-1: Programmed cell death protein 1
PD-L1: Programmed death-ligand 1
PI3K: Phosphoinositide 3-kinase
p53: Tumor protein p53
RBP: RNA-binding protein
ROC: Receiver operating characteristic
SD: Standard deviation
ssGSEA: Single-sample gene set enrichment analysis
SSZ: Sulfasalazine
STAT3: Signal transducer and activator of transcription 3
STITCH: Search Tool for Interactions of Chemicals
STRING: Search Tool for the Retrieval of Interacting Genes/Proteins
SVM-RFE: Support vector machine–recursive feature elimination
TAMs: Tumor-associated macrophages
TCGA: The Cancer Genome Atlas
TCGA-OV: TCGA ovarian serous cystadenocarcinoma project
TF: Transcription factor
TGF-β: Transforming growth factor beta
TIDE: Tumor Immune Dysfunction and Exclusion
TIL: Tumor-infiltrating lymphocyte
TIM-3: T-cell immunoglobulin and mucin-domain containing-3
TISIDB: Tumor and Immune System Interaction Database
TLR: Toll-like receptor
TNF: Tumor necrosis factor
Tregs: Regulatory T cells
