# Supplementary figures and images for: Reversing chemoresistance in ovarian cancer: network pharmacology reveals how hydroxychloroquine/sulfasalazine duotherapy remodels tumor inflammatory–immune microenvironment
Source: Front Immunol. 2026 Mar 26;17:1790210. doi: 10.3389/fimmu.2026.1790210 (PMC13062316; doi:10.3389/fimmu.2026.1790210)

**A**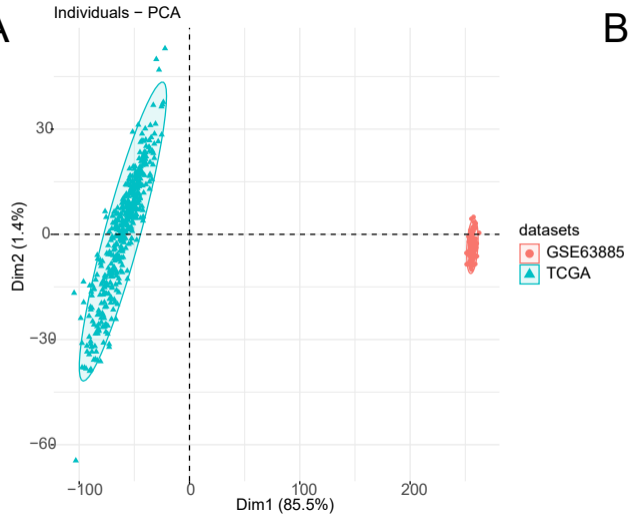**B**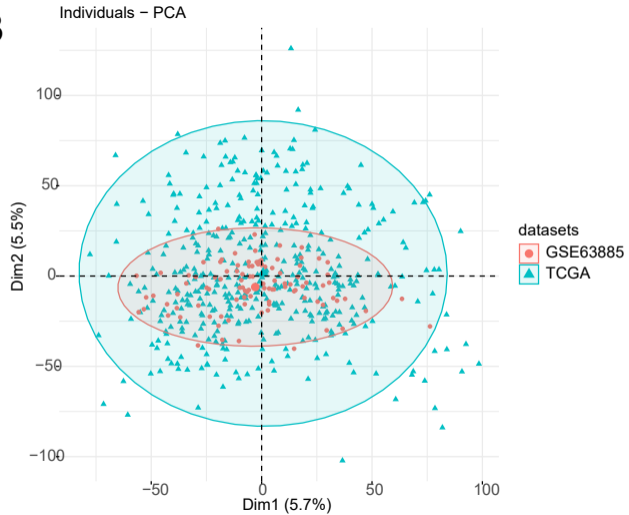

Supplement: Supplementary Figure 1 — PCA plots before and after data merging. PCA plots of TCGA-OV and GSE63885 before (A) and after (B) merging. [file DataSheet1.zip › Supplementary Files/Figure S1.pdf]

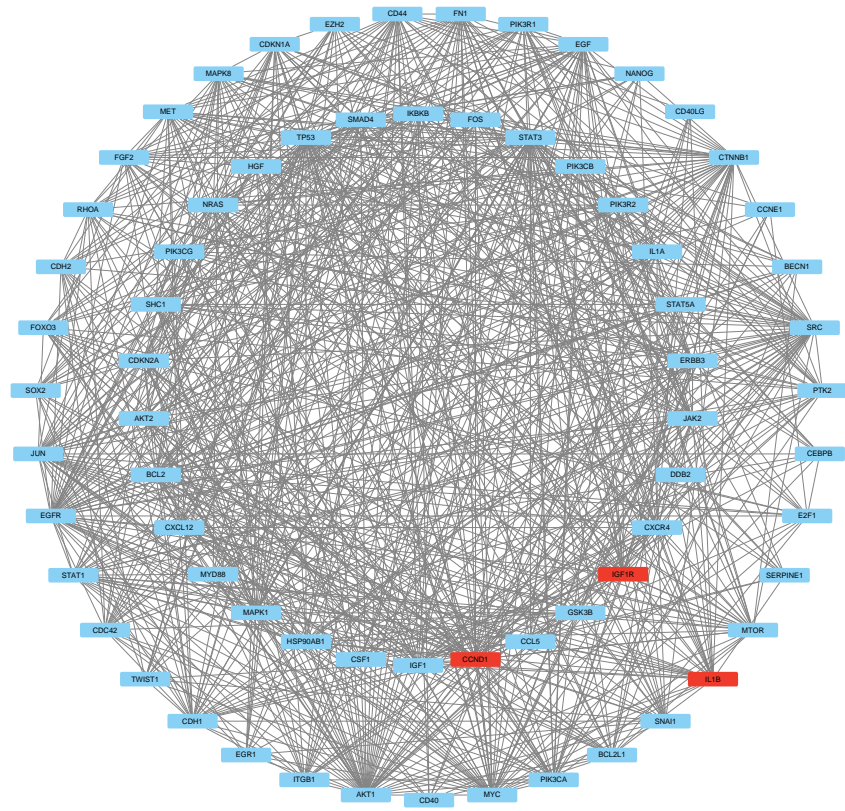

Supplement: Supplementary Figure 1 — PCA plots before and after data merging. PCA plots of TCGA-OV and GSE63885 before (A) and after (B) merging. [file DataSheet1.zip › Supplementary Files/Figure S2.pdf]

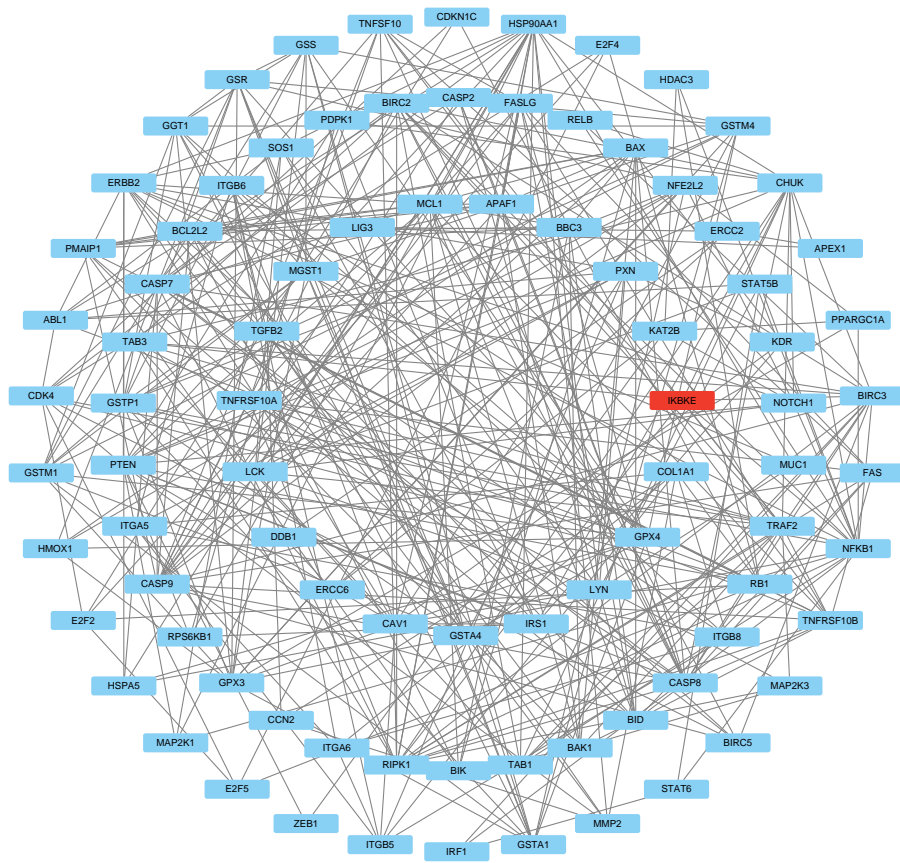

Supplement: Supplementary Figure 1 — PCA plots before and after data merging. PCA plots of TCGA-OV and GSE63885 before (A) and after (B) merging. [file DataSheet1.zip › Supplementary Files/Figure S3.pdf]

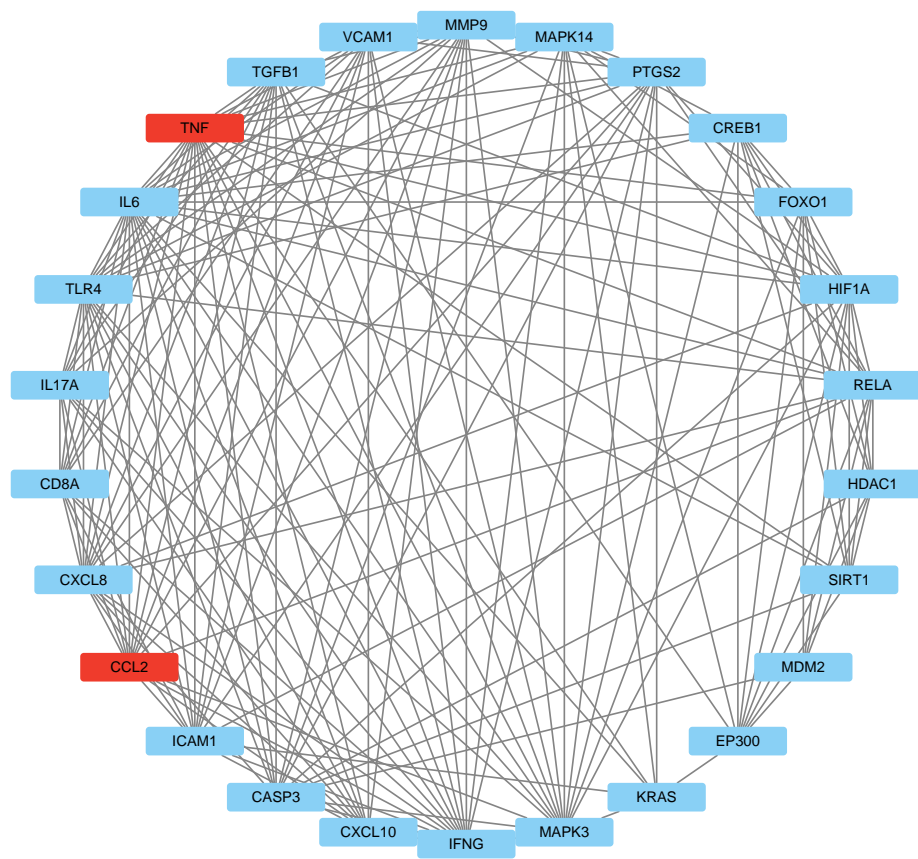

Supplement: Supplementary Figure 1 — PCA plots before and after data merging. PCA plots of TCGA-OV and GSE63885 before (A) and after (B) merging. [file DataSheet1.zip › Supplementary Files/Figure S4.pdf]
