# Supplementary material for: Reversing chemoresistance in ovarian cancer: network pharmacology reveals how hydroxychloroquine/sulfasalazine duotherapy remodels tumor inflammatory–immune microenvironment
Source: Front Immunol. 2026 Mar 26;17:1790210. doi: 10.3389/fimmu.2026.1790210 (PMC13062316; doi:10.3389/fimmu.2026.1790210)
Supplement: Supplementary file 2 [file Table1.docx]

**Supplementary Method 2.16: Cytokine panel and data handling**

Cytokine measurements were obtained as part of routine clinical testing and extracted from the hospital laboratory information system. The cytokine panel comprised 14 analytes: IL‑1β, IL‑2, IL‑4, IL‑5, IL‑6, IL‑8, IL‑10, IL‑12p70, IL‑17A, IL‑17F, IL‑22, IFN‑γ, TNF‑α, and TNF‑β. Concentrations were recorded in pg/mL. For values reported as below the assay reportable range (e.g., "<x"), a conservative single imputation was applied using half of the corresponding reporting threshold. Specifically, for the three cytokines used in the primary analyses, the reporting thresholds were IL‑6 < 1.5 pg/mL, IL‑8 < 1.0 pg/mL, and IL‑10 < 2.0 pg/mL, which were imputed as 0.75, 0.5, and 1.0 pg/mL, respectively.
